# How to spot a statistical problem: advice for a non-statistical reviewer

**DOI:** 10.1186/s12916-015-0510-5

**Published:** 2015-11-02

**Authors:** Darren C. Greenwood, Jennifer V. Freeman

**Affiliations:** Division of Epidemiology and Biostatistics, University of Leeds, Leeds, LS2 9JT UK

**Keywords:** Completeness of reporting, Editorial policy, Peer review, Presentation, Professional competence, Publishing, Recommendations to reviewers, Reporting guidelines, Statistics

## Abstract

Statistical analyses presented in general medical journals are becoming increasingly sophisticated. *BMC Medicine* relies on subject reviewers to indicate when a statistical review is required. We consider this policy and provide guidance on when to recommend a manuscript for statistical evaluation. Indicators for statistical review include insufficient detail in methods or results, some common statistical issues and interpretation not based on the presented evidence. Reviewers are required to ensure that the manuscript is methodologically sound and clearly written. Within that context, they are expected to provide constructive feedback and opinion on the statistical design, analysis, presentation and interpretation. If reviewers lack the appropriate background to positively confirm the appropriateness of any of the manuscript’s statistical aspects, they are encouraged to recommend it for expert statistical review.

## Introduction

Most papers published in general medical journals, including *BMC Medicine*, contain some element of statistical methods, analysis and interpretation. There is evidence that statistical analyses are becoming increasingly sophisticated [1]. Expert statistical review has therefore become an integral part of the editorial process. Some journals send all manuscripts for statistical review. Other journals only send a manuscript for statistical review if it is considered necessary; for example, if the methods are particularly complex or if the Editor or subject reviewer has concerns. The approach taken by *BMC Medicine* is to ask subject reviewers if they are able to assess all the statistical aspects of the manuscript themselves or whether they recommend an additional statistical review.

One potential weakness of this approach is that it is a system that relies heavily upon the statistical expertise of subject reviewers, who may not have a formal qualification or professional accreditation in statistics. As such, the subject reviewer may be competent in a specific range of statistical methods applicable to their area of expertise, but may not necessarily be aware of more general statistical issues or more recent methodological developments and best practices. The subject reviewer may be able to spot the most egregious errors but is likely to miss the subtlety of inappropriate statistics that might be picked up by an appropriately qualified statistical expert. The aim of this paper is to provide subject reviewers with some help in deciding when a manuscript might benefit from undergoing a proper statistical review. Our comments mainly refer to review of primary research, rather than to systematic reviews and meta-analysis, for which a separate tutorial is available [2].

Statistical review is an important element of the peer-review process that has been shown to substantially improve the quality of manuscripts [3–5]. This relates not only to the statistical analysis, but also to other relevant areas, such as data sources, study design, presentation of results and interpretation of results [1, 6].

We argue that sending a paper for statistical review should not be limited to studies where the subject reviewer considers the methods to be potentially incorrect, or beyond their expertise. Rather, the subject reviewer should generally recommend expert statistical review unless they can positively confirm that there are no problems with the study design, statistical analysis, presentation and interpretation of results.

Although some statistical irregularities are subtle and only likely to be detected by a statistical expert, subject reviewers should consider some of the following indicators of the more common problems encountered in primary research:

### Is there sufficient detail to review the statistical aspects?

- Have the relevant reporting guidelines been followed (for example, CONSORT for randomized controlled trials [7] or STROBE for observational studies [8])?
- Have the authors justified their sample size and made reasonable assumptions about the effect size they consider important to detect? Have they presented enough information to verify their calculations [9]?
- Have the methods been provided in sufficient detail to replicate the results if the data were available [1, 10, 11]?
- Is it clear how all the results were derived, such as the test or model used, including any covariates, and were the assumptions made in implementing the model reasonable?

### Are there any common statistical issues?

- Are there lots of *P* values, or subgroup analyses, particularly unplanned subgroup analyses that were not pre-specified, indicating multiple testing [12]?
- Are the covariates adjusted for in models appropriate, without remaining confounding, or over-adjustment for covariates on the causal pathway (for example, longitudinal studies where a covariate is measured after the exposure)?
- Are there any hierarchical data structures (for example, cluster randomized trials, repeated measures or matching of cases and controls), and if so has the analysis taken this into account?
- Should the analysis address agreement rather than association [13]?
- Has the intention-to-treat principle been appropriately applied in pragmatic effectiveness trials [14, 15]?
- Have continuous variables been categorized? Have trends been ignored? This may not necessarily mean an inappropriate analysis, but may indicate that a full statistical review would be beneficial.

### Is the presentation of results appropriate?

- Is there any evidence of selective reporting? Do the main results focus on the main research question, or do they deviate to a secondary question or subgroup? This is particularly problematic if the subgroup analysis was not specified prior to undertaking the analysis [12].
- Are results presented without estimates, just *P* values [16]?
- Are estimates presented with no confidence intervals? Standard errors alone are rarely adequate for presenting the uncertainty in estimates, either in the text or graphically [16].

### Is the interpretation of results appropriate?

- Are limitations of observational studies correctly acknowledged, with no implication of causality in the wording of results and conclusions?
- Are results over-extrapolated, beyond the range of the data, or to populations not represented by the study sample?
- Is there an appropriate consideration of the impact of any incomplete or missing data?

Although there might be alternative approaches to statistical analysis or presentation, this does not necessarily imply the authors’ methods are invalid. What is important is that the methods chosen are appropriate for the research question and have been done correctly [17]. *BMC Medicine* allows comments under “discretionary revisions” where such observations can be made.

The same caution we recommend to non-statistical reviewers also applies to statistical experts. Statistical methods are many and varied, particularly in a general medical journal such as *BMC Medicine*. Some of the more specialist methods may be outside of the experience of a general statistical reviewer. Consequently they should be encouraged to recommend that the editorial office approach an additional specialist in those particular methods for further scrutiny of the article.

## Conclusions

In advising the Editor on publication, reviewers are required to comment on whether a manuscript is methodologically sound and clearly written. Within that context, they are expected to provide clear, constructive feedback and opinion on study design, statistical analysis, presentation and interpretation of results. We have provided a number of indicators to assist the non-statistical reviewer in this task. If reviewers lack the appropriate background to positively confirm the appropriateness of any of the manuscript’s statistical aspects, they are encouraged to recommend it for expert statistical review.

## Abbreviations

CONSORT: Consolidated Standards of Reporting Trials
STROBE: Strengthening the Reporting of Observational Studies in Epidemiology
